# Sources and Determinants of Vitamin D Intake in Danish Pregnant Women

**DOI:** 10.3390/nu4040259

**Published:** 2012-04-16

**Authors:** Camilla B. Jensen, Sesilje B. Petersen, Charlotta Granström, Ekaterina Maslova, Christian Mølgaard, Sjurdur F. Olsen

**Affiliations:** 1 Centre for Fetal Programming, Department of Epidemiology Research, Statens Serum Institut, 5, Orestads Boulevard, DK 2300 Copenhagen S, Denmark; Email: ssp@ssi.dk (S.B.P.); cgs@ssi.dk (C.G); sfo@ssi.dk (S.F.O.); 2 Departments of Nutrition and Epidemiology, Harvard School of Public Health, Boston, MA 02115, USA; Email: emaslova@hsph.harvard.edu; 3 Department of Human Nutrition, Copenhagen University, 30, Rolighedsvej, DK 1958 Frederiksberg C, Denmark; Email: cm@life.ku.dk

**Keywords:** vitamin D, pregnancy, sources, diet, supplements, determinants, socio-demography, gestation, lifestyle

## Abstract

Vitamin D deficiency during pregnancy has been associated with the development of several adverse health outcomes, e.g., pre-eclampsia, gestational diabetes mellitus, preterm delivery, low birth weight, birth length, and bone mineral content. The aims of the present study were to estimate the intake and sources of vitamin D in Danish pregnant women and to examine potential determinants of vitamin D intake of the recommended level (10 µg per day). In 68,447 Danish pregnant women the mean ± SD for vitamin D intake was 9.23 ± 5.60 µg per day (diet: 3.56 ± 2.05 µg per day, supplements: 5.67 ± 5.20 µg per day). 67.6% of the women reported use of vitamin D supplements but only 36.9% reported use of vitamin D supplements of at least 10 µg. Supplements were the primary source of vitamin D for the two higher quartiles of total vitamin D intake, with diet being the primary source for the two lower quartiles. Determinants of sufficient total vitamin D intake were: high maternal age, nulliparity, non-smoking, and filling out of the Food Frequency Questionnaire (FFQ) during summer or fall. We propose that clinicians encourage vitamin D supplementation among pregnant women, with special focus on vulnerable groups such as the young, smokers and multiparous women, in order to improve maternal and fetal health both during and after pregnancy.

## 1. Introduction

Vitamin D is a pre-hormone that occurs naturally in a limited number of foods and which can be synthesized in the skin when the skin is exposed to sunlight [1]. The need for vitamin D can be fulfilled by exposing skin to sunlight; however, in northern latitudes the sun exposure only synthesizes vitamin D from April to October [1] and vitamin D must be obtained from other sources during the winter period. Sun exposure may also increase the risk of skin cancer and it may therefore be problematic to encourage sun exposure as the only source of vitamin D [2]. Dietary vitamin D intake is very limited and therefore supplementation is needed to avoid vitamin D deficiency during sun-deprived periods. 

During pregnancy, vitamin D is transported from mother to fetus through the placenta in the form of 25(OH)D which is also the established marker of vitamin D status [1,3,4]. At birth, there is a correlation between maternal and child vitamin D concentrations, where the concentration in the umbilical cord blood is around 80% of maternal blood concentration [4,5]. This suggests that vitamin D deficiency in pregnancy will lead to more children being born deficient [5]. 

In the unborn child, vitamin D is important for fetal bone development through its role in regulating calcium homeostasis [6,7]. Studies have suggested that children born to women with low vitamin D status have lower scores in terms of birth weight, birth length, and bone mineral content [4]. Other studies propose an association between vitamin D deficiency and pre-eclampsia [8]. Studies have also suggested that vitamin D sufficiency has a protective effect against pre-term delivery and gestational diabetes mellitus through its immunomodulatory and anti-inflammatory properties [9]. Animal studies show a possible association between maternal vitamin D status and brain development in pups, which could possibly play a role in cognitive and neurological development [4]. Other suggested consequences of vitamin D deficiency during pregnancy are increased risks of: schizophrenia, type 1 diabetes, multiple sclerosis, heart disease, cancer, and small for gestational age diagnosis [10,11,12,13]. 

The definition of optimal vitamin D status and optimal intake level of vitamin D have been subject to much debate, and recommendations vary between countries. In Scandinavia, all pregnant women are recommended 10 µg of vitamin D per day to ensure sufficient vitamin D status [14]. Vitamin D intake of 10 µg per day is expected to ensure 25(OH)D concentration of 25 nmol/L or more [14,15]. In the United States and Canada the recommended dietary allowance is 15 µg per day [16]. Several studies have reported that vitamin D intake among pregnant women is lower than recommended. For instance in Norway, 63% of pregnant women consumed <10 µg per day of vitamin D [17], in Finland the prevalence was 85% [18], and 50% in the United States [19]. 

The Danish National Birth Cohort (DNBC) offers information on vitamin D intake in Danish pregnant women as well as information on socio-demographic and lifestyle factors, and is thereby an excellent opportunity to investigate potential determinants of vitamin D intake. This information can help identify women at risk of insufficient vitamin D intake and potentially vitamin D deficiency. This would ensure that attention is focused on the risk groups and could aid in improvement of maternal and fetal health during pregnancy. 

The aims of the present study, conducted within the Danish National Birth Cohort, were: (1) to estimate the vitamin D intake among Danish pregnant women; (2) to investigate the sources of vitamin D; and (3) to examine potential determinants of sufficient total vitamin D intake among Danish pregnant women, including gestational, lifestyle, and dietary factors. 

## 2. Materials and Methods

### 2.1. The Danish National Birth Cohort

**Figure 1:**
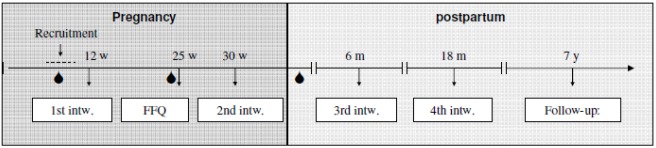
Timing of activities in the Danish National Birth Cohort study. 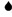
—blood draw.

Data was derived from the Danish National Birth Cohort (DNBC), a nationwide prospective cohort study with long term follow-up. The cohort enrolled 101,042 pregnancies across Denmark in the time period from 1996 to 2002. Eligibility for enrolment in the study was pregnancy in the given time period, ability to fill in questionnaires, and take part in interviews in Danish. Invitations to the cohort study were administered by general practitioners who distributed recruitment forms and information about the cohort during the first antenatal visit. During the study, the women filled out a recruitment form, a food frequency questionnaire (FFQ), participated in four telephone interviews, and donated two blood samples during pregnancy and one blood sample from the umbilical cord at delivery (Figure 1). Information on lifestyle, diet, and socioeconomic status of the pregnant women was obtained from these activities. Follow-up of the cohort is still ongoing [20]. After exclusion of participants with missing information on dietary vitamin D intake, our study population consisted of 68,447 pregnancies from all over Denmark. Women were allowed to participate in the cohort with more than one pregnancy. 

### 2.2. Diet

Dietary data was obtained from a semi-quantitative FFQ that included 360 items. The FFQ was mailed to the women when they were in gestational week 25 and covered food intake during the previous month. For analysis purposes, all food frequencies were transformed into times per day. The daily frequencies were multiplied with standard portion sizes to calculate food intake in grams. The food intake in grams was then coupled with the Danish Food Tables [21]. For calculation purposes, standard recipes were created when dealing with dishes containing multiple ingredients. Nutrient content was corrected for nutrient loss during cooking [22]. The calculation of nutrient intake was performed in FoodCalc (software) [23]. 

### 2.3. Vitamin D Supplements

Vitamin D intake from supplements was calculated based on questions asking about supplements in the FFQ and in DNBC interviews 2 and 3. The FFQ provided detailed information on daily supplement use during the four weeks prior to filling out the FFQ. Interviews 2 and 3 provide information on supplement use on a weekly basis in gestational weeks 1 to 30 and 30 to birth, respectively. A database of all known dietary supplements in Denmark was created listing their nutrient content, based on information from the “Danish Veterinary and Food Administration”. The database is not validated but validation of a similar database from the Norwegian birth cohort (MoBa) showed good agreement between supplementary vitamin D measured by FFQ and 25(OH)D-levels [24]. For every woman, the frequency of each supplement reported in the FFQ or the interviews were combined with the given supplement in the database and the daily intake was calculated on the basis of the recommended daily intake of the supplement. All missing responses were assigned null values (17,447 observations in the FFQ) [25,26]. These calculations led to the creation of a data set with information for all participants (*N* = 68,447) on mean daily vitamin D intake from supplements calculated from the FFQ and mean daily vitamin D intake from supplements per gestational week from the interviews. 

### 2.4. Other Factors

Information on maternal and lifestyle characteristics was obtained from DNBC interviews 1 and 2. The following factors were regarded as potential determinants of vitamin D intake and included in the analyses: maternal age (<20, 20–25, 25–30, 30–35, 35–40, ≥40), pre-pregnancy BMI (≤18.5, 18.5–25, 25–30, 30–35, 35–40, >40), energy intake, parity (0, 1, 2, 3+), civil status (single, coupled/married), socio-occupational status (high, medium, skilled, student, unskilled, unemployed), physical activity level (inactive, light, moderate, high), smoking during pregnancy (non-smoker, occasional smoker, <15 cigarettes per day, ≥15 cigarettes per day), alcohol consumption during pregnancy (yes, no), season of filling out the FFQ, and planned pregnancy (planned, partly planned, not planned).

### 2.5. Statistical Analyses

Comparison of total energy intake and dietary intakes for sufficient *vs*. insufficient total vitamin D intake was performed by *t*-test for normally distributed continuous variables. For variables not normally distributed, we used Wilcoxon signed-rank test. Univariate and multivariate logistic regression was employed to estimate the association between total vitamin D intake and socio-demographic, gestational, and life-style characteristics. All subjects with missing information on any of the variables in the regression models were left out of the analyses. The values were missing at random. Odds ratios and 95% confidence intervals were estimated. *P* < 0.05 was considered as significant. All statistical analyses are performed in SAS for Windows version 9.3. (SAS Institute Inc., Cary, North Carolina). 

## 3. Results

Mean dietary vitamin D intake was 3.56 ± 2.05 µg per day, mean vitamin D intake from supplements was 5.67 ± 5.20 µg per day, and mean total intake of vitamin D was 9.23 ± 5.60 µg per day. The highest total intake was 80.68 µg per day but only four women had intakes ≥50 µg per day, which is considered the upper limit of intake that is unlikely to cause adverse health effects. 44.4% had sufficient total vitamin D intake. 

**Table 1:** Intake of vitamin D supplements in the Danish National Birth Cohort during gestational weeks 21–25 (*N* = 68,447) *.

Daily vitamin D dose		N (%)
0 µg		22,214 (32.5)
Women who took vitamin D supplements		46,233 (67.5)
	>0 µg and ≤2.5 µg	1020 (1.5)
	>2.5 µg and <5 µg	2110 (3.1)
	≥5 µg and <7.5 µg	16,710 (24.4)
	≥7.5 µg and <10 µg	1114 (1.6)
	≥10 µg	25,279 (36.9)

* Based on information from the food frequency questionnaire.

**Figure 2:**
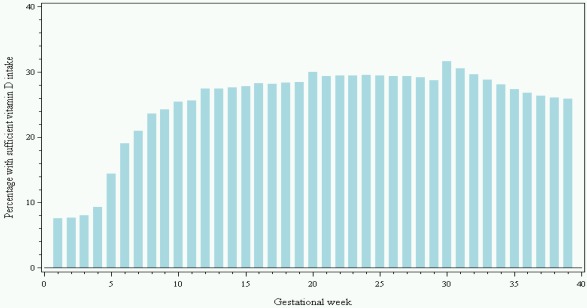
Proportion of women (%) reporting intake of vitamin D supplements ≥10 µg per day per gestational week in gestational week 1 to 39 based on information from DNBC interviews 2 and 3 (*N* = 68,447).

67.6% of participants reported use of vitamin D supplements in any dose (Table 1) and 36.9% reported use of vitamin D supplements of at least 10 µg, which is the dose now recommended during pregnancy by *The Danish National Board of Health*. The most common dose was 10 µg per day (31.5%) followed by 5 µg per day (22.2%) (data not shown). Figure 2 shows the proportion of women reporting use of vitamin D supplements ≥10 µg per day per gestational week based on the results from DNBC interview 2 and 3. From gestational week 7 onwards, more than 20% of the women reported use of vitamin D supplements ≥10 µg per day with a maximum proportion of 31.7% in gestational week 30. In gestational week 20 to 25 (the period also covered by the FFQ), the proportion of women reporting use of vitamin D supplements ≥10 µg per day was approximately 30%. The proportion of women reporting use of vitamin D supplements ≥10 µg per day found in the FFQ was of 36.9%. From gestational week 30, the proportion of women who reported sufficient use of vitamin D supplements decreased. 

In order to investigate the sources of vitamin D, total vitamin D intake was divided into quartiles, and mean intake of dietary vitamin D and vitamin D from supplements in each quartile was calculated. The sources were ranked according to their contribution to total vitamin D intake (Table 2), and we found that supplements were the primary source of vitamin D for the two higher quartiles and diet was for the two lower quartiles.

**Table 2:** Mean intake of vitamin D from diet, supplements and total intake, along with ranking of sources according to quartiles of total vitamin D intake (*N* = 68,447) *.

	Quartiles of total vitamin D intake (µg/day)							
	1		2		3		4	
Mean vitamin D intake	µg/day (%)	Order	µg/day (%)	Order	µg/day (%)	Order	µg/day (%)	Order
Total	2.62 (100)		6.82 (100)		11.20 (100)		16.29 (100)	
Supplements	0.03 (1.15)	2	3.31 (48.53)	2	7.88 (70.36)	1	11.46 (70.35)	1
Diet	2.59 (98.85)	1	3.51 (51.47)	1	3.33 (29.73)	2	4.83 (29.65)	2

* Based on information from the food frequency questionnaire covering intake in gestational weeks 21–25.

**Table 3:** Mean intake of vitamin D from food groups according to quartiles of dietary vitamin D intake in gestational weeks 21–25 (*N* = 68,447) *.

	Quartiles of dietary vitamin D intake (µg/day)							
	1		2		3		4	
Mean vitamin D intake	µg/day (%)	Order	µg/day (%)	Order	µg/day (%)	Order	µg/day (%)	Order
All food groups	1.7 (100)		2.6 (100)		3.7 (100)		6.3 (100)	
Pork	0.2 (11.8)	3	0.2 (7.7)	4	0.2 (5.4)	4	0.2 (3.2)	3
Beef/veal	0.2 (11.8)	3	0.2 (7.7)	4	0.2 (5.4)	4	0.2 (3.2)	3
Processed meat	0.1 (5.9)	4	0.1 (3.8)	5	0.1 (2.7)	5	0.1 (1.6)	4
Fish and seafood	0.4 (23.5)	1	0.9 (34.6)	1	1.8 (48.6)	1	4.3 (68.3)	1
Egg	0.2 (11.8)	3	0.4 (15.4)	3	0.4 (10.8)	3	0.5 (7.9)	2
Low fat dairy	0.3 (17.6)	2	0.5 (19.2)	2	0.5 (13.5)	2	0.5 (7.9)	2

* Based on information from the food frequency questionnaire.

The dietary intake of vitamin D from different food groups for each quartile of dietary vitamin D intake is presented in Table 3. Thirty-seven food groups were analyzed, but only 14 contributed with vitamin D. The food groups cheese, whole fat dairy products, margarine, poultry, butter, yogurt, dressing/sauce, and lamb contributed amounts <0.1 µg per day. Intakes below 0.1 µg might be subject to uncertainty and therefore these were not considered to be contributing to dietary vitamin D intake. The main sources of dietary vitamin D for all quartiles were fish and seafood, followed by low fat dairy products and eggs. The higher the dietary vitamin D intake, the higher amount of vitamin D consumed from fish and seafood. The same was true for low fat dairy products and eggs; however, the increase in vitamin D was very small. The contribution of vitamin D from pork, beef/veal, and processed meat was the same for all quartiles of dietary vitamin D intake. 

We wanted to examine whether the women with sufficient total vitamin D intake differed from the women with insufficient total vitamin D intake (Table 4). A statistically-significant direct association was seen between sufficient total vitamin D intake and maternal age. Adjustment for pre-pregnancy BMI, parity, civil status, socio-occupational status, physical activity level, season of filling out the FFQ, smoking and alcohol use during pregnancy, total energy intake, and planning of pregnancy made the direct association between maternal age and sufficient total vitamin D intake stronger. A statistically-significant inverse association was seen between sufficient total vitamin D intake and parity and number of cigarettes daily. Inclusion of the above-mentioned covariates in the analyses made the association between sufficient total vitamin D intake and number of children stronger, but had no effect on the association between sufficient total vitamin D intake and number of smoked cigarettes during pregnancy. Single women had lower odds ratio (OR) for sufficient total vitamin D intake than married/coupled women, however, this relation was not statistically significant. Before adjustment for relevant covariates, the OR for sufficient total vitamin D intake was significantly lower for partly planned or non-planned pregnancies than for planned pregnancies. The OR for sufficient total vitamin D intake was significantly higher for mothers filling out the FFQ during summer or fall. This association was weakened a little by adjustment for relevant covariates.

**Table 4:** Determinants of sufficient total vitamin D intake (≥10 µg per day) *vs.* insufficient total vitamin D intake (<10 µg per day) (*N* = 58,763) *.

			Sufficient total vitamin D intake		
		*N*	OR (95% CI) crude	OR (95% CI) adjusted **	*P* ***
**Age**					<0.0001
	Age < 20	829	0.8 (0.7, 0.9)	0.7 (0.6, 0.8)	
	20 ≤ Age < 25	10,237	1.0 (1.0, 1.1)	0.9 (0.9, 1.0)	
	25 ≤ Age < 30	26,518	1.0 (ref.)	1.0 (ref.)	
	30 ≤ Age < 35	16,852	0.9 (0.9, 0.9)	1.1 (1.0, 1.1)	
	35 ≤ Age < 40	4053	0.9 (0.9, 1.0)	1.2 (1.1, 1.3)	
	Age ≥ 40	274	1.1 (0.9, 1.4)	1.4 (1.1, 1.8)	
**Pre-pregnancy BMI**					0.1
	BMI ≤ 18.5	2463	1.1 (1.0, 1.2)	1.1 (1.0, 1.2)	
	18.5 < BMI ≤ 25	40,311	1.0 (ref.)	1.0 (ref.)	
	25 < BMI ≤ 30	11,334	1.0 (0.9, 1.0)	1.0 (1.0, 1.1)	
	25 < BMI ≤ 30	3394	1.0 (1.0, 1.1)	1.1 (1.0, 1.2)	
	25 < BMI ≤ 30	926	1.0 (0.8, 1.1)	1.0 (0.9, 1.2)	
	BMI > 40	335	1.1 (0.9, 1.3)	1.1 (0.9, 1.4)	
**Parity**					<0.0001
	0	28,894	1.0 (ref.)	1.0 (ref.)	
	1	21,150	0.7 (0.7, 0.7)	0.6 (0.6, 0.7)	
	2	7194	0.6 (0.5, 0.6)	0.5 (0.5, 0.5)	
	3+	1525	0.5 (0.4, 0.6)	0.4 (0.4, 0.5)	
**Civil status**					0.7
	Single	1006	0.9 (0.8, 1.1)	1.0 (0.8, 1.1)	
	Coupled/married	57,757	1.0 (ref.)	1.0 (ref.)	
**Socio-occupational status**					0.6
	High	5818	1.0 (ref.)	1.0 (ref.)	
	Medium	17,196	1.0 (0.9, 1.1)	1.0 (0.9, 1.1)	
	Skilled	11,010	1.0 (0.9, 1.0)	1.0 (0.9, 1.1)	
	Student	5547	1.0 (0.9, 1.1)	1.0 (0.9, 1.1)	
	Unskilled	13,286	0.9 (0.9, 1.0)	1.0 (0.9, 1.1)	
	Unemployed	5906	0.9 (0.8, 0.9)	1.0 (0.9, 1.1)	
**Physical activity**					0.6
	Inactive	36,171	1.0 (ref.)	1.0 (ref.)	
	Light	13,220	1.1 (1.0, 1.1)	1.0 (1.0, 1.1)	
	Moderate	8260	1.1 (1.0, 1.1)	1.0 (0.9, 1.0)	
	Moderate	1112	1.0 (0.9, 1.2)	0.9 (0.8, 1.0)	
**Smoking during pregnancy**					<0.0001
	Nonsmoker	44,561	1.0 (ref.)	1.0 (ref.)	
	Occasional smoker	7466	1.1 (1.0, 1.1)	1.0 (1.0, 1.1)	
	<15 cigarettes per day	5725	0.8 (0.8, 0.9)	0.8 (0.8, 0.9)	
	>15 cigarettes per day	1011	0.7 (0.6, 0.8)	0.7 (0.6, 0.8)	
**Alcohol during pregnancy**					0.04
	No	23,466	1.0 (ref.)	1.0 (ref.)	
	Yes	35,297	1.0 (1.0, 1.1)	1.0 (1.1)	
**Planned pregnancy**					0.03
	Planned	45,354	1.0 (ref.)	1.0 (ref.)	
	Partly planned	7350	0.9 (0.9, 0.9)	0.9 (0.9, 1.0)	
	Not planned	6059	0.9 (0.8, 0.9)	1.0 (0.9, 1.0)	
**Season**					<0.0001
	Winter	13,490	1.0 (ref.)	1.0 (ref.)	
	Spring	15,431	1.1 (1.0, 1.1)	1.0 (1.0, 1.1)	
	Summer	15,930	1.5 (1.4, 1.6)	1.5 (1.4, 1.6)	
	Fall	13,912	1.3 (1.2, 1.3)	1.2 (1.2, 1.3)	

* Based on information from the food frequency questionnaire and DNBC interview 1 and 2; ** Adjusted for maternal age, pre-pregnancy BMI, parity, civil status, socio-occupational status, physical activity level, season of filling out the FFQ, smoking and alcohol use during pregnancy, total energy intake, planning of pregnancy; *** *P*-value for trend in the adjusted model.

Dietary patterns of women with sufficient *vs*. insufficient total intake of vitamin D are shown in Table 5.

**Table 5:** Intakes of energy, macronutrients and food groups among women with sufficient total vitamin D intake (≥10 µg per day) *vs*. insufficient total vitamin D intake (<10 µg per day) in gestational weeks 21–25 (*N* = 68,447) *.

	Sufficient total vitamin D intake (*N* = 30,357)	Insufficient total vitamin D intake (*N* = 38,090)	*P*-value
Total energy (KJ)	10,042.6 (2439.8)	9852.0 (2365.4)	<0.0001 ^†^
Protein (% of energy)	15.2 (2.4)	15.0 (2.3)	0.002 ^†^
Carbohydrate (% of energy)	54.5 (5.8)	54.3 (5.9)	0.1 ^†^
Fat (% of energy)	29.8 (5.9)	30.3 (6.0)	0.03 ^†^
Alcohol (% of energy)	0.27 (0, 0.6)	0.29 (0, 0.7)	<0.0001 ^‡^
Dietary variables (g/MJ)			
Dairy products	606.2 (359.7, 842.4)	611.4 (366.1, 846.8)	0.02 ^‡^
Fruit	130.5 (67.5, 231.9)	116.6 (65.2, 222.1)	<0.0001 ^‡^
Vegetables	113.2 (75.1, 168.2)	102.7 (67.0, 152.8)	<0.0001 ^‡^
Meat	72.7 (31.1)	72.9 (31.1)	0.9 ^†^
Fish	17.6 (9.3, 29.7)	14.6 (7.9, 24.2)	<0.0001 ^‡^
Poultry	24.0 (13.2, 35.3)	19.4 (11.2, 31.4)	<0.0001 ^‡^
Cereals	24.4 (5.5, 48.0)	23.6 (5.2, 48.4)	0.2 ^‡^
Potatoes	115.9 (80.7, 165.8)	120.1 (82.7, 173.0)	<0.0001 ^‡^
Fats	18.9 (11.1, 29.6)	20.1 (11.5, 31.4)	<0.0001 ^‡^

Data are presented as mean (standard deviation) or median (25th percentile, 75th percentile). g/MJ: gram per megajoule. * Based on information from the food frequency questionnaire; ^†^ *t*-test; ^‡^ Wilcoxon signed rank test.

## 4. Discussion

Dietary vitamin D intake was very low in the study population. None of the participants reported dietary intakes that match the recommended intake level in pregnancy of 10 µg per day, which underlines the necessity of vitamin D supplementation. The recommendations for pregnant women in most northern countries include a vitamin D supplement of 10 µg per day. In the DNBC, 67.6% of the participants reported use of vitamin D supplements, but only 36.9% used supplements with a vitamin D dose of 10 µg or more per day. When the study was conducted there were no formal recommendations from *the Danish National Board of Health *on use of vitamin D supplements during pregnancy, and both the number of users and the supplement dose may therefore be higher today. 

We found that women started to use supplements relatively late in pregnancy, but when they started they continued throughout pregnancy. The late start might indicate the point when the women realized that they were pregnant. The most common doses of vitamin D in supplements were 10 µg and 5 µg, indicating that the available supplements in Denmark all have doses of 5 and 10 µg vitamin D. Low dose supplements make it difficult for the women to obtain the recommended dose of vitamin D. All vitamin D supplements aimed at pregnant women should therefore contain 10 µg. 

When looking at the total vitamin D intake in the DNBC we found that as many as 55.6% had insufficient intakes even though 67.6% report use of vitamin D supplements in some dose. 11.8% have intakes below the lower limit (2.5 µg), which is considered the level below which an intake could lead to deficiency symptoms. This has also been seen in the Norwegian Mother and Child Cohort study (MoBa), where 81.4% report use of some type of supplements, 63% did not reach the recommended intake level, and as many as 12% had intake levels <2.5 µg [17]. In a Finnish pregnancy study 74% of vitamin D among supplement users came from supplements, but only 15% reached the recommended intake level of 10 µg per day [27]. 

The main source of vitamin D was supplements for the two higher quartiles of total vitamin D intake, and diet for the two lower quartiles. Both absolute dietary vitamin D and vitamin D from supplements increased with quartile of total vitamin D intake. This suggests that the women who have vitamin D-containing diets are also the ones who use high dose supplements. We found supplements to contribute substantially to vitamin D intake, but a large group of women still did not reach the recommended level of intake. Public attention must be focused on vitamin D supplementation in order to improve public health. 

We found a lower likelihood of sufficient total vitamin D intake among young, smoking, multiparous women. In other studies, maternal age was also found to be inversely associated with vitamin D intake and the use of supplements, suggesting that younger individuals may be less conscious about health matters [17,18,27,28,29]. Multiparous women were found to be less likely to have sufficient total vitamin D intakes compared to nulliparous women. One other study also found this association [29]. In the Norwegian birth cohort MoBa parity was inversely associated with supplement use [17]. This association was not seen in a Finnish birth cohort [27]. Since multiparous women have experienced pregnancy before it is possible that they are less concerned about their pregnancy, and thus also less conscious about the need of supplementation. We found that smoking was inversely associated with vitamin D intake. Other studies also found that non-smokers had higher intakes of vitamin D [19,29] and that non-smokers were more likely to use vitamin D supplements [17,27]. Smokers might be overall less health conscious and therefore not aware of the importance of using vitamin D supplements in pregnancy. In other studies, overweight women have been found to be less likely to have sufficient vitamin D intake and to use supplements [17,27,29]. We did not see this association. High socio-economic status and education was found to be directly associated with vitamin D intake and supplement use in other studies [17,18,27,28,29]. This difference was not seen in our study. As expected, pregnancy-planners were more likely to have sufficient total vitamin D intake, probably because they were more conscious about their pregnancy. However, the association was not significant after adjustment. We also found civil status to be non-significantly associated with vitamin D intake, with single women being less likely to have sufficient total vitamin D intake. This association was not seen in other studies which might indicate that the difference we saw may have been random. We found a significant association between season and vitamin D intake of at least 10 µg per day where filling out the FFQ during summer or fall had higher ORs than filling out the FFQ during spring or winter. It is questionable whether this association is random or if it reflects a seasonal variation in dietary vitamin D intake or in the awareness of the importance of vitamin D supplementation.

Women with sufficient total vitamin D intake were found to have significantly higher intakes of total energy, protein, fruit, vegetables, poultry and fish, and significantly lower intakes of fat, dairy products, and potatoes, compared to women with insufficient total vitamin D intake. Among Finnish pregnant women, it was found that consumption of fruit, berries, and fish were more common among supplement users, and consumption of margarine was less common [27]. Together, these results suggest that women who use vitamin D supplements or have sufficient total vitamin D intake might have a healthier diet than women with insufficient vitamin D intake or who do not use supplements. Even though the differences in diet between women with sufficient *vs.* women with insufficient total vitamin D intake are statistically significant, it is possible that the differences are clinically trivial, since the estimates in absolute numbers only differ very little. 

A database of all known supplements in Denmark has been developed in order to estimate vitamin D intake from supplements. This database has not been validated yet and it is therefore uncertain if the estimated intake reflects the real intake. Some analytical data suggest that actual levels of nutrients exceed labeled levels but according to the US Department of Agriculture, the information on vitamin D deviation from labels has not yet been estimated [30,31]. However, the database was based on information from “the Danish Veterinary and Food Administration”, which continuously performs random checks of all approved supplements, and therefore we are confident that the database contains valid information. Also, validation of a similar database from the Norwegian birth cohort MoBa showed good agreement between supplementary vitamin D measured by FFQ and 25(OH)D levels [24].

The proportion of women who used supplements of 10 µg or more per day estimated from the interviews was only 31.7%, which is similar to, but lower than, the proportion estimated from the FFQ (36.9%). The missing responses on supplement use were assigned null values as is done in other studies [25,26]. It is possible that null value imputation has caused an underestimation of vitamin D supplementation, but since the estimated use of vitamin D supplements is similar to the one seen in Norway [17], and lower than the one in Finland [27], we do not think this issue is relevant. 

The DNBC study is one of the largest birth cohorts that has assessed dietary intake in pregnancy, and the cohort offers a wide range of information on lifestyle, gestational, and socio-demographic factors. In cohort studies, generalizability is a potential problem and it may arise at recruitment or during follow-up. Approximately 35% of all pregnant women in Denmark were enrolled in the DNBC, and the women who were enrolled are likely to be more concerned about health matters in relation to pregnancy, compared to the background population [32]. Vitamin D intake in this study might therefore not be representative for the background population. However, we believe that the determinants of vitamin D intake seen in this study may adequately reflect the ones of the background population. Determinants of vitamin D insufficiency must be taken into consideration in making recommendations on vitamin D intake in pregnancy, to include more vulnerable groups that may be at higher risk of deficiency.

## 5. Conclusions

55.6% of the DNCB participants report insufficient total vitamin D intake, even though 67.6% report use of vitamin D supplements. We found supplements to contribute substantially to vitamin D intake. The main source of vitamin D was supplements for the two higher quartiles of total vitamin D intake, and diet for the two lower quartiles. Determinants of sufficient total vitamin D intake were high maternal age, giving birth during fall or winter, nulliparity, and low number of daily cigarettes use during pregnancy. We propose that clinicians should encourage vitamin D supplementation among pregnant women, with a special focus on vulnerable groups, such as younger, smoking and multiparous women, in order to improve maternal and fetal health both during and after pregnancy. 
